# Quantitative Assessment of Microbial Pathogens and Indicators of Wastewater Treatment Performance for Safe and Sustainable Water Reuse in India

**DOI:** 10.1128/spectrum.01720-22

**Published:** 2022-10-31

**Authors:** Shruti Chowdhari, Shubham Rana, Samridhi Rana, Christina M. Morrison, Sarah Elizabeth Abney, Rajveer Singh, Patrick L. Gurian, Amit Kumar, Arun Kumar, Walter Q. Betancourt, Perumal Vivekanandan

**Affiliations:** a Kusuma School of Biological Sciences, Indian Institute of Technology Delhi, New Delhi, India; b Department of Civil Engineering, Malaviya National Institute of Technology, Jaipur, Rajasthan, India; c Department of Civil Engineering, Indian Institute of Technology, Delhi, India; d Department of Civil, Architectural and Environmental Engineering, Drexel University, Philadelphia, Pennsylvania, USA; e Water & Energy Sustainable Technology (WEST) Center, University of Arizona, Tucson, Arizona, USA; Health Canada

**Keywords:** microbial indicators, molecular quantitation, human pathogens, wastewater treatment performance, PMMoV, *Giardia*

## Abstract

Currently, there is no data on the molecular quantification of microbial indicators of recycled water quality in India. In this study, multiple microbial pathogens and indicators of water quality were evaluated at three wastewater treatment plants located in two Indian cities (New Delhi and Jaipur) to determine the treatment performance and suitability of recycled water for safe and sustainable reuse applications. Real-time polymerase chain reaction (PCR) was used for the rapid evaluation of six human pathogens and six microbial indicators of fecal contamination. Among the microbial indicators, pepper mild mottle virus (PMMoV), F^+^RNA-GII bacteriophage, Bacteroides thetaiotamicron, and four human pathogens (Norovirus genogroups I & II, Giardia, and Campylobacter coli) were detected in all of the influent samples analyzed. This work suggests that the raw influents contain lower levels of noroviruses and adenoviruses and higher levels of Giardia compared to those reported from other geographic regions. Overall, the efficacy of the removal of microbial targets was over 93% in the final effluent samples, which is consistent with reports from across the world. PMMoV and Giardia were identified as the best microbial targets, from the microbial indicators spanning across bacteria, bacteriophages, DNA/RNA viruses, and protozoan parasites, by which to evaluate treatment performance and recycled water quality in Indian settings, as they were consistently present at high concentrations in untreated wastewater both within and across the sites. Also, they showed a strong correlation with other microbial agents in both the raw influent and in the final effluent. These findings provide valuable insights into the use of culture-independent molecular indicators that can be used to assess the microbial quality of recycled water in Indian settings.

**IMPORTANCE** Wastewater treatment plants (WWTPs) have rapidly increased in India during the last decade. Nonetheless, there are only a few labs in India that can perform culture-based screening for microbial quality. In the last 2 years of the pandemic, India has witnessed a sharp increase in molecular biology labs. Therefore, it is evident that culture-independent real-time PCR will be increasingly used for the assessment of microbial indicators/pathogens in wastewater, especially in resource-limited settings. There is no data available on the molecular quantitation of microbial indicators from India. There is an urgent need to understand and evaluate the performance of widely used microbial indicators via molecular quantitation in Indian WWTPs. Our findings lay the groundwork for the molecular quantitation of microbial indicators in WWTPs in India. We have screened for 12 microbial targets (indicators and human pathogens) and have identified pepper mild mottle virus (PMMoV) and Giardia as the best molecular microbiological indicators in Indian settings.

## INTRODUCTION

While wastewater reuse is a promising approach to meet the increasing demand for water, it is vital to assess the microbiological quality of the recycled water for safe reuse applications. Untreated municipal wastewater harbors numerous enteric pathogens, including those of human fecal origin (bacteria, protozoa, viruses) that are capable of causing mild to severe enteric diseases (1). Thus, untreated wastewater and improperly treated recycled water for landscape irrigation or for the irrigation of food crops pose significant human health risks.

The total wastewater generated by urban settlements in India in the year 2020 was estimated to be 26.4 km^3^, of which only 7.38 km^3^ (28%) is treated by 1,093 sewage treatment plants (STPs) located in different parts of the country (2). The urban local bodies (ULBs) and regulatory bodies address water reuse for the irrigation of edible and nonedible food crops (including horticulture), noncontact impoundments, washing, and industrial activities (2). Evaluation methods for the efficiency of wastewater treatment processes currently cover physicochemical parameters, whereas a framework for the estimation of microbial removal efficiency is currently under development.

It is well-documented that exposure to sewage-polluted water can lead to disease outbreaks associated with enteric viruses. For example, a localized outbreak of acute gastroenteritis in southern Mumbai in 2006 was associated with the cocirculation of multiple enteric virus strains that were primarily spread via the consumption of sewage contaminated drinking water (3). In addition, a waterborne rotavirus outbreak during post-earthquake Kashmir in 2005 was associated with poor sanitary conditions that resulted in the sewage contamination of drinking water (4). Cases of pediatric diarrhea due to cryptosporidiosis and giardiasis have also been associated with the consumption of untreated groundwater in a few districts in India (5, 6).

Studies from different geographic locations have demonstrated the occurrence of human enteric pathogens, including Cryptosporidium, Giardia, and numerous enteric viruses in treated wastewater effluents (7–12). In general, most of the literature available on monitoring strategies for the microbial removal efficiency of wastewater treatment facilities is from developed countries. There is a dearth of data on the efficacy of microbial indicators/pathogen removal from wastewater from developing countries, including India.

Traditionally, the microbial quality of recycled water for reuse in India is based on the measurement of turbidity or suspended solids, plus other process parameters (including chemical oxygen demand [COD], biological oxygen demand [BOD_5_], ammonia removal, etc.), along with the monitoring of fecal indicator bacteria (FIB) via total coliform, fecal coliform, and fecal *Streptococci* counts (13, 14). Numerous studies have demonstrated the limitations of bacterial indicators to evaluate the occurrence and persistence of viral and protozoan pathogens in wastewaters and thus underscore the need for specific indicators for different groups of pathogens (15–18). Both viruses and protozoan parasites have demonstrated a greater resistance to UV light disinfection than traditional microbial indicators have (15, 19, 20). In addition, viruses show variable nonlinear removal in comparison to conventional indicator organisms during the treatment (21, 22). Relatively fewer groups of viruses have been identified as etiological agents of waterborne acute gastroenteritis. This is not because of their infrequent occurrence but because of a lack of specific diagnostics. In order to ensure minimal risk of exposure to fecally derived enteric pathogens from recycled water, standard methods and microbial indicators are required to evaluate the distribution and occurrence of pathogens in wastewater in specific geographic locations.

The monitoring of hundreds of human-pathogenic enteric viral strains known to contaminate aquatic systems is not practical. Thus, nonpathogenic indicators and human pathogens that are relatively resistant to processes used in wastewater treatment are quantitated in influents and effluents to assess log reduction values and the overall microbial quality of the wastewater. In addition to the enumeration of the fecally shed microbial species, the discrimination of the source of the fecal contamination (i.e., human or nonhuman source) in water before its reuse is important in order to reduce the exposure of the human population to potential human pathogens. To this end, monitoring nonpathogenic viruses that are commonly shed in human feces, such as PMMoV (a plant virus of the Virgaviridae family and the Tobamovirus genus), has provided valuable information as a microbial target of treatment performance (23). PMMoV has been found in a high percentage of pepper-based food products and as the dominant RNA virus in human feces by several metagenomic studies (24). In addition, male F-specific coliphages (MSC) or the direct detection of human-pathogenic enteric viruses, such as enteroviruses and adenovirus, have been demonstrated to be reliable tools with which to assess human sewage pollution in water (25, 26).

Culturing may be the best method to assess infectivity; however, the availability of the necessary expertise and infrastructure for virus culturing may be a major limiting factor in resource-limited settings. Over the last 2 years, there has been an unprecedented increase in the infrastructure and manpower training for real-time PCR across the world, including in developing and underdeveloped countries, to help facilitate the diagnosis of SARS-CoV-2 in the ongoing coronavirus disease pandemic. In other words, contrary to the limited number of facilities for virus culturing, real-time PCR expertise and infrastructure is now widely available throughout the world (27). It is therefore evident that real-time PCR will be increasingly used for the assessment of microbial indicators/pathogens in wastewater, especially in resource-limited settings. We therefore sought to investigate nonpathogenic microbial indicators of human fecal contamination and human pathogens from influents and effluents in three wastewater treatment plants in India.

In this study, a comparative quantitative analysis of putative microbial indicators of fecal contamination, including PMMoV, F-specific RNA coliphages genogroup I to IV, and B. thetaiotaomicron (*B. theta*), as well as human pathogens, including human adenoviruses (HAdVs), enteroviruses (EV), noroviruses (NoV GI-II), Giardia, and C. coli, was conducted at three municipal wastewater treatment plants (WWTPs) located in two major cities in northern (Delhi) and north-western (Jaipur) India. The microbial indicators selected for this study have been consistently reported in the influents and effluents of wastewater treatment facilities across the world with a moderate to high statistical correlation between the loads of the different microbial targets (nonpathogenic indicators and human pathogens). Finally, this study aimed to identify a nonpathogenic indicator and a human pathogen that could serve as reliable indicators of treatment performance and of the overall water quality of treated effluents intended for water reuse in Indian settings.

## RESULTS

### Molecular detection and quantification of microbial indicators and pathogens in the raw influent.

**FIG 1:**
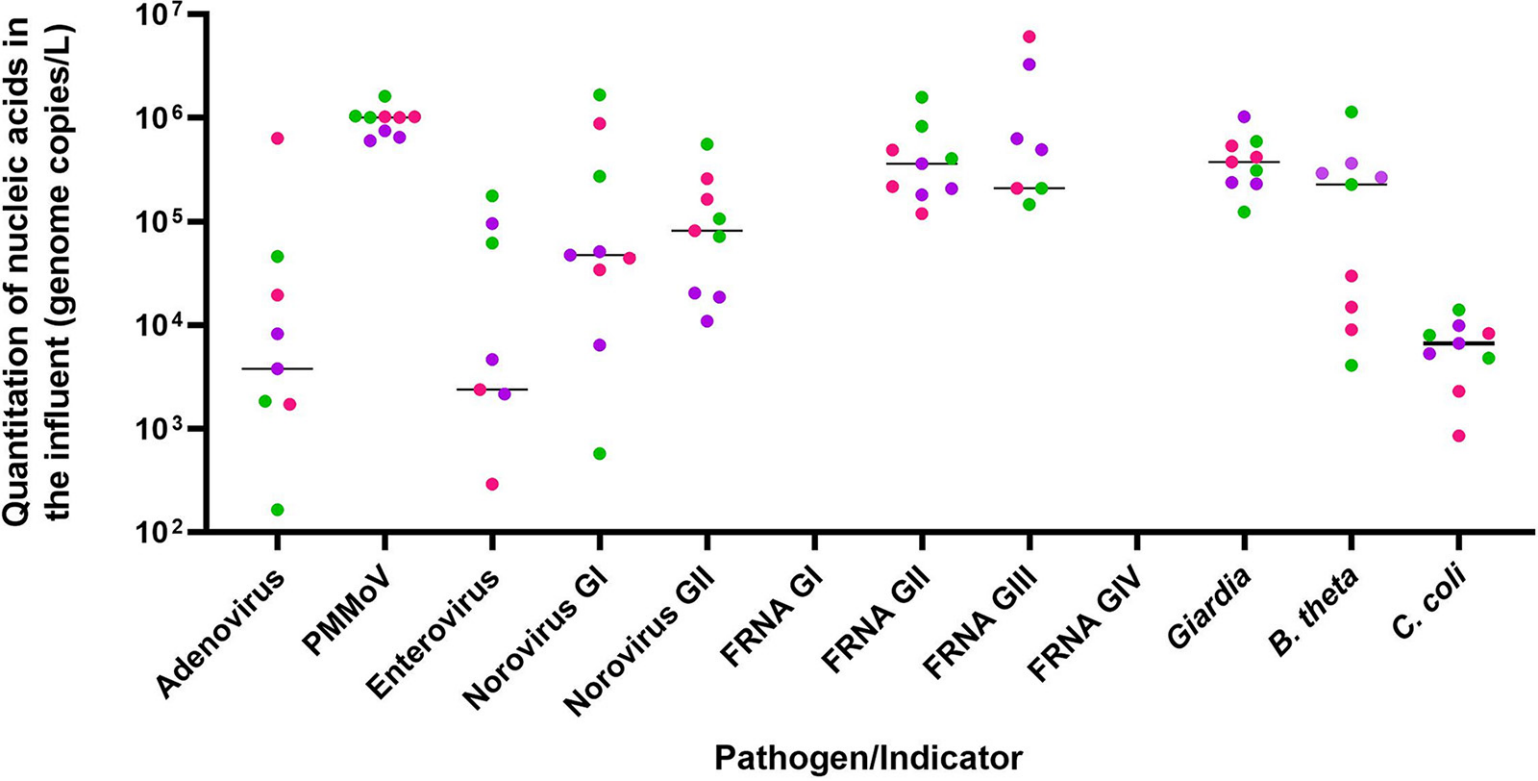
Molecular quantification of microbial indicators and pathogens in influent samples. Quantitation of nucleic acids from indicators of microbial contamination and from human pathogens (genome copies/L) across influent wastewater samples (*n* = 9). The green, pink, and purple dots represent samples from WWTP-1, WWTP-2, and WWTP-3, respectively. The horizontal line represents median concentrations. Microbial indicators or human pathogens that are not detected are not plotted in this graph.

All of the microbial pathogens and indicators were detected in influent samples at slightly different frequencies (Table 1). The limit of detection was optimized to ≤10 genome copies/reaction for all of the PCR assays. The following 7 indicators were detected in 100% of the influent samples: (i) the 3 nonpathogenic indicators of human fecal contamination (i.e., PMMoV, fecal coliform bacteriophage F^+^RNA GII, and *B. theta*) and (ii) four human pathogens (i.e., NoV GI, NoV GII, Giardia, and C. coli). Among the influent samples (*n* = 9), PMMoV was the most abundant microbial target, ranging from 5.7 to 6.2 log_10_ gc/L across the influents (*n* = 9) with a coefficient of variance (CV) of 29.4%, followed by F^+^RNA GII (5.0 to 6.1 log_10_ gc/L, CV of 89.5%) and *B. theta* (3.6 to 6.0 log_10_ gc/L, CV of 129.8%). F^+^RNA GIII was detected in 77.7% of the influent samples (5.3 to 6.7 log_10_ gc/L, CV of 133.5%), while F^+^RNA GI and F^+^RNA GIV were not detectable in any of the influents (Fig. 1).

**TABLE 1:** Detection of nucleic acids from microbial indicators and human pathogens in raw influent samples^*a*^

Pathogen/Indicator	Pathogen/indicator characteristics	Host characteristics	Positive samples/no. tested	WWTP-1	WWTP-2	WWTP-3
Adenovirus	Nonenveloped dsDNA viruses	Numerous mammals, including humans	88.8% (8/9)	+ + +	+ + +	+ + –
PMMoV	Nonenveloped (+)ssRNA viruses	Pepper plants	100% (9/9)	+ + +	+ + +	+ + +
Enterovirus	Nonenveloped (+)ssRNA viruses	Numerous mammals, including humans	77.7% (7/9)	+ – +	+ – +	+ + +
Norovirus GI	Nonenveloped (+)ssRNA viruses	Numerous mammals, including humans	100% (9/9)	+ + +	+ + +	+ + +
Norovirus GII	Nonenveloped (+)ssRNA viruses	Numerous mammals, including humans	100% (9/9)	+ + +	+ + +	+ + +
F^+^RNA GI	Nonenveloped (+)ssRNA viruses	Faecal coliform bacteria	0% (0/9)	− − −	− − −	− − −
F^+^RNA GII	Nonenveloped (+)ssRNA viruses	Faecal coliform bacteria	100% (9/9)	+ + +	+ + +	+ + +
F^+^RNA GIII	Nonenveloped (+)ssRNA viruses	Faecal coliform bacteria	77.7% (7/9)	+ − +	+ − +	+ + +
F^+^RNA GIV	Nonenveloped (+)ssRNA viruses	Faecal coliform bacteria	0 % (0/9)	− − −	− − −	− − −
Giardia	Protozoan parasite, dsDNA	Numerous mammals, including humans	100% (9/9)	+ + +	+ + +	+ + +
B. thetaiotaomicron	Gram-negative bacteria, dsDNA	Exclusively found in the human gut	100% (9/9)	+ + +	+ + +	+ + +
*C. coli*	Gram-negative bacteria, dsDNA	Commensals in animals, causes foodborne illness in humans	100% (9/9)	+ + +	+ + +	+ + +

a G: Genogroup. “+” indicates that the microbial indicator/pathogen was detected, and “−” indicates that the microbial indicator/pathogen was not detected. Three sampling events are represented for each microbial indicator/pathogen at all three of the sites.

PMMoV has been detected in both domestic wastewater influents and effluents in a concentration range between 1 to 9 log_10_ gc/L in different parts of the world. PMMoV RNA has remarkable stability in wastewater, making it an attractive indicator of human fecal contamination in domestic wastewaters (28). In addition to being detected at the highest concentrations in our study, PMMoV was associated with the lowest coefficient of variance, suggesting minimal variations within and across sites. Thus, PMMoV may represent the most suitable indicator for human fecal contamination in urban settings in India.

Bacteriophages are widely distributed in the environment and are known to exist for all known bacterial species (29). Enteric bacteriophages (male specific F^+^RNA coliphages) have been found in both wastewaters and various foods (including meat, such as fresh chicken, ground beef, pork sausage, and shellfish) due to their resistance to harsh environmental conditions and to chemical and physical treatments, such as disinfection, compared to fecal indicator bacteria (e.g., coliforms and enterococci). F^+^RNA coliphages have been well-recognized as the model organisms in the characterization and management of water resources for nearly 5 decades (29–32). Among the four antigenically distinct genogroups (G) of F^+^RNA coliphages, G I and G IV are found in animal fecal waste, whereas G II and G III are predominately associated with human feces or wastewater derived from domestic sewage. Therefore, the genotype-specific detection of F^+^RNA coliphages in wastewater is a promising tool for tracking the sources (animal or human) of fecal contamination (33, 34). The Bacteroides-associated marker Bacteroides thetaiotaomicron (*B. theta*) has been described as a symbiont and a “niche” organism in the human gut (35), and its presence in wastewater is a presumptive bacterial indicator of human fecal contamination (36). In our study, we were able to detect F^+^RNA GII and GIII, whereas F^+^RNA GI and GIV were not detectable in any of our influent samples, indicating that the source of fecal waste is primarily derived from human excrement.

Among the pathogens that were consistently detected in all of the raw influent samples, Giardia was the most abundant in the influent samples (5.1 to 6.0 log_10_ gc/L, CV of 59.3%), followed by NoV GII (4.0 to 5.7 log_10_ gc/L, CV of 114.7%), NoV GI (2.7 to 6.2 log_10_ gc/L, CV of 161.9%), and C. coli (2.9 to 4.1 log_10_ gc/L, CV of 56.2%). HAdV (2.2 to 5.8 log_10_ gc/L, CV of 230.8%) and EV (2.4 to 5.2 log_10_ gc/L, CV of 127.6%) were detected in 88.8% and 77.7% of the raw influent samples, respectively (Fig. 1).

Of note, the norovirus and adenovirus loads in our influent samples (median loads for noroviruses GI and GII were < 5 log_10_ gc/L, and median loads for adenoviruses were well below 4 log_10_ gc/L) were lower than those reported from other geographical regions (norovirus loads and adenovirus loads in influent samples were up to 8 log_10_ or 9 log_10_ gc/L) (11, 37). The preliminary evaluation of virus recovery efficiency using MS2 and ΦX 174 bacteriophages spiked into tertiary effluent and untreated wastewater indicated >80% recoveries obtained via dead-end ultrafiltration (DEUF) using Ashai Kasei REXEED-25S ultrafilters (unpublished data). Since there is no data on noroviruses and adenoviruses that have been reported from India, it is particularly challenging to understand the lower loads for these viruses in our samples. A meta-analysis has revealed that the prevalence of norovirus infections may be lower in many developing countries compared to that observed in developed countries (38). In addition, fish and shellfish consumption per capita in India is among the lowest in the world (39). Seasonal variations have been documented among enteric adenoviruses with peaks in spring, summer, and midwinter (40). Our samples were collected during the fall, and perhaps this may explain, in part, the lower adenovirus loads in our influent samples. Regardless of the underlying reasons, the lower loads for noroviruses and adenoviruses in our influent samples suggests that these viruses may not be useful indicators of virus reductions during wastewater treatment in India.

It has been well-documented that the shedding of enteroviruses in feces (i.e., number shed per gram of feces) is about 3 to 6 log_10_ lower than that of noroviruses and adenoviruses (15). In our study, the load for enteroviruses at WWTP-1 (in Delhi) was comparable with that reported from other geographic regions (41, 42). However, the enterovirus loads in the influents from WWTP-2 and WWTP-3 in Jaipur were lower. This finding suggests the existence of significant region-specific variations in enterovirus concentrations at the same time points between Delhi and Jaipur. This finding also suggests that enteroviruses may not be ideal surrogates for human pathogens in wastewater in India.

The concentration of Giardia varied from undetectable up to approximately 5 log_10_ gc/L in raw wastewater samples from around the world (43). Interestingly, in the present study, Giardia was consistently detected at much higher levels (>5 log_10_ gc/L in 8 of the 9 samples of raw influents) in the influent samples, suggesting that it may be a good microbial marker for pathogens in Indian settings. Campylobacter coli was detected at a variable concentration range of 2.9 to 4.1 log_10_ gc/L across all of the influents.

The physicochemical parameters, including the pH, temperature, turbidity, BOD_5_, COD, and total Kjeldahl nitrogen (TKN) of the collected influents and treated effluents from the WWTPs are summarized in the Supplemental Material (Table S1). The percentage reduction in BOD5, COD, and TKN in the effluent wastewater samples from all three of the WWTPs is also summarized in the Supplemental Material (Fig. S1).

### Quantitation of microbial indicators and pathogens in effluent samples.

**FIG 2:**
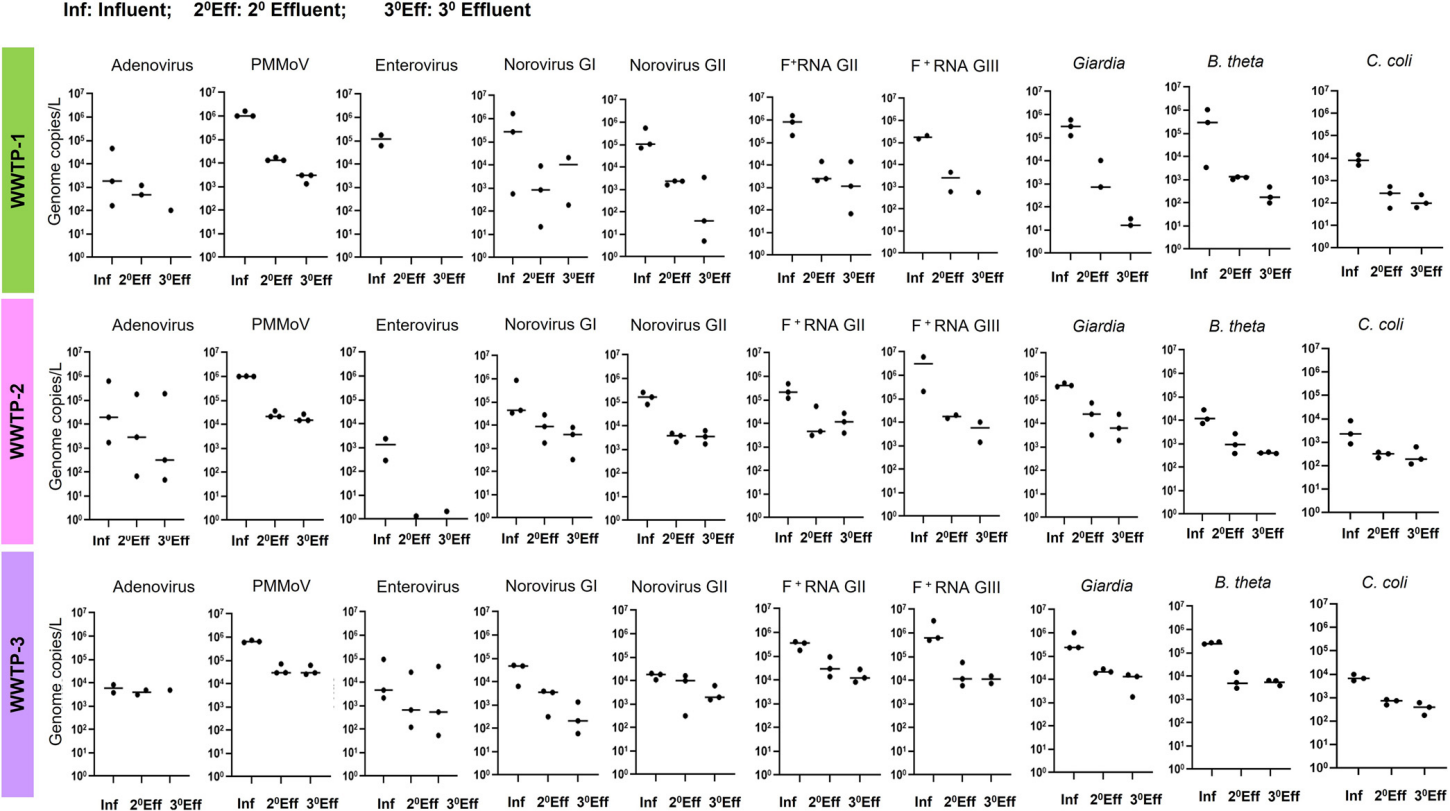
Comparison of microbial loads in influent and effluent samples. Concentration (genome copies/L) of each microbe (including human adenoviruses [HAdV], pepper mild mottle virus [PMMoV], enteroviruses [EV], norovirus genogroup 1 [NoV GI], norovirus genogroup 2 [NoV GII], Giardia, and F-specific RNA coliphages genogroups I and III [F+RNA GII, F^+^RNA GIII], *B. theta*, and C. coli) at different stages of treatment (Inf, influent; 2° Eff, 2° effluent; and 3° Eff, 3° effluent) within a WWTP. Each data point represents the concentration (genome copies/L) of the respective microbial indicator/pathogen. The horizontal line represents the median values.

**FIG 3:**
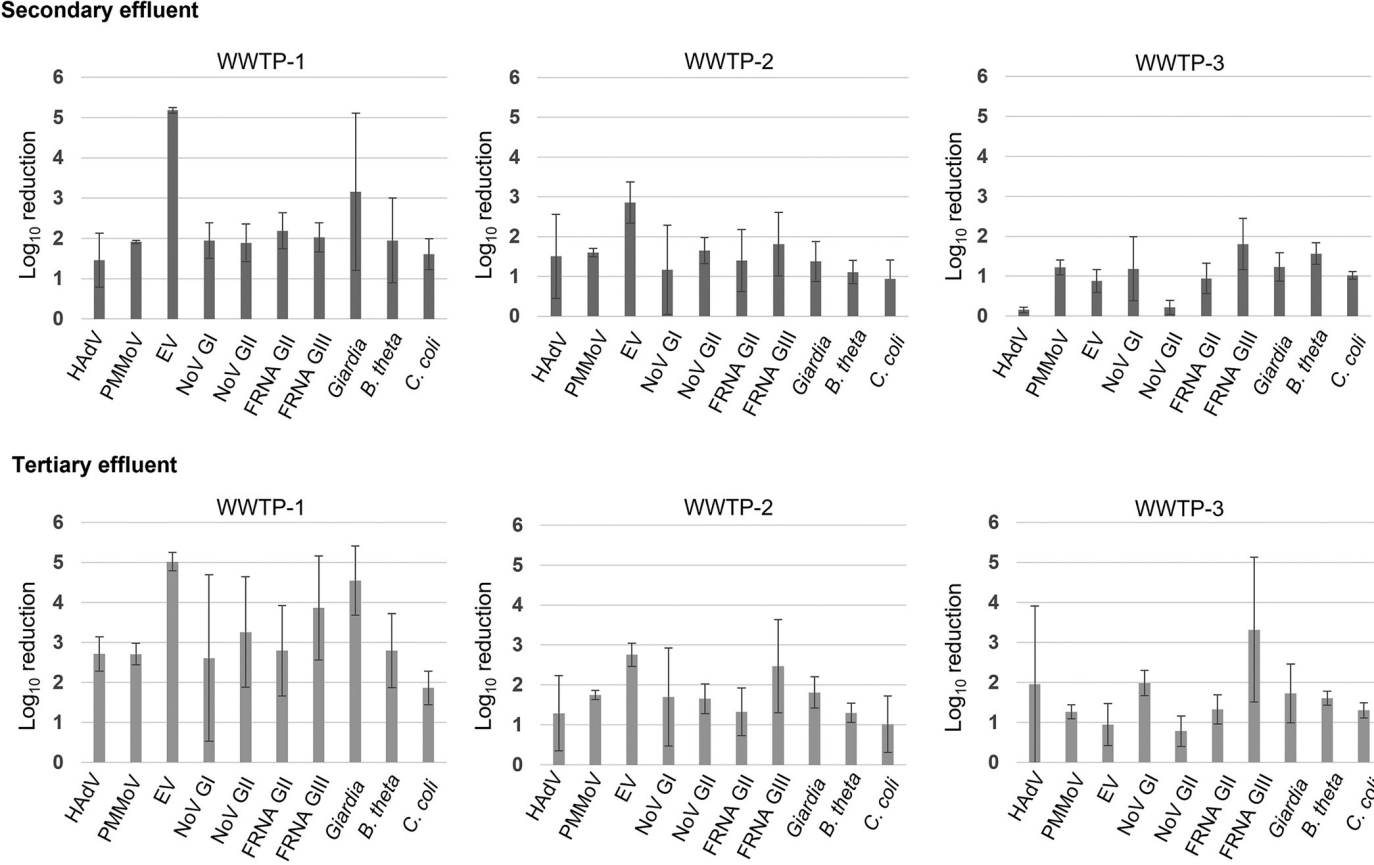
Log_10_ reduction of microbial indicators and pathogens. Bar graphs represent a log_10_ reduction in nucleic acids (mean ± standard deviation [SD]) in the secondary (*n* = 3, upper panel) and tertiary (*n* = 3, lower panel) effluents with respect to the influents within each WWTP.

The majority of the microbial indicators and pathogens detected in the influent samples were also detected after treatment at lower concentrations than those observed in the influent samples. As shown in Fig. 2 (concentration of microbial targets at various stages of treatment) and Fig. 3 (log reduction values [LRV] of microbial targets after secondary and tertiary treatment), among the pathogenic enteric viruses, a 5 log_10_ reduction of EV was consistently observed at WWTP-1.

HAdV and norovirus GI were detected at variable concentrations before and after tertiary treatment at each WWTP. For HAdV, a reduction (mean LRV [±SD]) of 2.7 (±0.4), 1.2 (±0.9), and 1.9 (±1.9) log_10_ was achieved within WWTP-1, -2, and -3, respectively. For NoV GI, a reduction of 2.6 (±2.0), 1.7 (±1.2), and 1.9 (±0.3) log_10_ was achieved within WWTP-1, -2, and -3, respectively. On the other hand, the reduction of Norovirus GII corresponded to 3.2 (±1.3), 1.6 (±0.3), and 0.7 (±0.3) log_10_ for WWTP-1, -2, and -3, respectively. PMMoV was detected at a consistently high concentration across all of the influent samples collected from the three WWTPs. The reduction in PMMoV ranged from 1.2 (±0.17) to 2.7 (±0.2) log_10_ across the WWTPs. Moreover, the PMMoV levels did not vary much across effluent samples from either within or between sites (Fig. 2). PMMoV has been frequently found in treated effluents from different countries (10, 44, 45) and in recharged groundwater for reuse applications (22). As a plant virus, PMMoV has an extremely high stability in both terrestrial and aquatic environments. This virus has been shown to survive standard food processing, such as in the manufacturing of hot chili sauce and powdered chili (28). The thermal inactivation of this and other members of the *Tomabovirus* genus occurs above 80°C.

F^+^RNA GII showed a maximum but variable reduction in the effluents from WWTP-1 and somewhat lower but consistent reductions in effluents from WWTP-2 and -3. The F^+^RNA GIII showed a reduction of 3.8 (±1.2), 2.4 (±1.1), and 3.3 (±1.8) log_10_ in WWTP-1, -2, and -3, respectively. For Giardia, a reduction of 4.5 (±0.8), 1.8 (±0.3), and 1.7 (±0.7) log_10_ was observed in the tertiary effluents from WWTP-1, -2, and -3, respectively.

**FIG 4:**
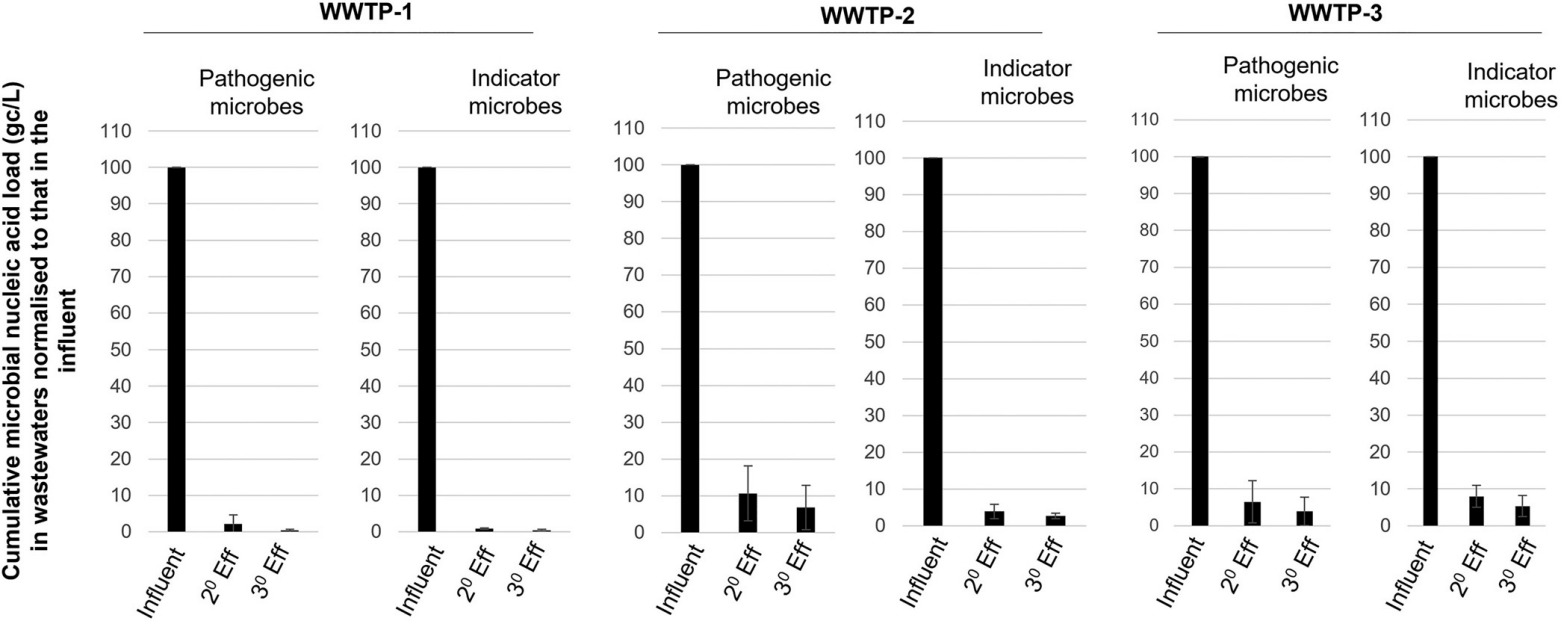
Percentage reduction of microbial indicators and pathogens. Reduction in microbial nucleic acid load in the secondary and tertiary (final) effluent, normalized to the load in the influent, for pathogens (noroviruses GI and GII, enteroviruses, human adenoviruses, C. coli and Giardia) as well as nonpathogenic indicators of human fecal contamination (i.e., PMMoV, fecal coliform bacteriophage F^+^RNA GI-IV and *B. theta*). Each bar represents the mean of three sampling events ± the SD from each WWTP.

The reduction in load of the final effluent, normalized to the influent load, for pathogens and nonpathogenic microbial indicators of human fecal contamination (i.e., PMMoV, fecal coliform bacteriophage F^+^RNA GII, and *B. theta*) is shown in Fig. 4. The extent of the removal of the microbial indicators/pathogens ranged from about 93.2% to 99.6% (i.e., from 1.7 log_10_ to 2.9 log_10_). Several reports from developed countries indicate a 75% to 99% reduction of loads in final effluents compared to those observed in influent samples (21, 46). Our data suggest that the extent of reduction in the microbial load in the studied WWTPs are comparable. In addition, our results suggests that nonpathogenic microbial indicators of human fecal contamination are associated with increased retention in the final effluent compared to molecular indicators of human pathogens. This finding reiterates that the detection of nonpathogenic indicators of human fecal contamination is more reliable than the detection of pathogens, not only because of their abundance but also because of their enhanced resistance to routinely used wastewater treatment processes.

Studies have compared the quantitative PCR (qPCR) data with classical culture-based methods for the quantification of microbial indicators in wastewater and found a strong correlation between the two methods (47–49). Furthermore, F^+^ specific coliphages range from 5.0 to 7.0 log_10_ PFU/L in raw municipal wastewater samples from different countries (31). This range is comparable to the qPCR data obtained for the F^+^ specific coliphages (5.0 to 6.7 log_10_ gc/L) in the wastewater influents sampled in this study. Nonetheless, several studies report an increased sensitivity or overestimation by qPCR compared to plaque assay methods. For example, higher levels of F^+^-specific coliphages were detected by using qPCR compared to those measured using plaque assays (47, 50). Culture-based methods detect only viable or infectious microbes, whereas qPCR detects genomes from both viable and nonviable microbes. In addition, the differences in viability and the resistance to wastewater treatment among microbial indicators/pathogens will impact the correlation between culture-based methods and qPCR. Culture-based methods may be ideal to assess the public health threat posed by a pathogen. Nonetheless, culture-based methods are not available for many microbial indicators/pathogens (e.g., Giardia, PMMoV, and noroviruses). Therefore, apart from data from culture-based methods, qPCR data have also been used for quantitative microbial risk assessment (QMRA) (51, 52).

### Correlation between the microbial loads of indicators and pathogens.

**FIG 5:**
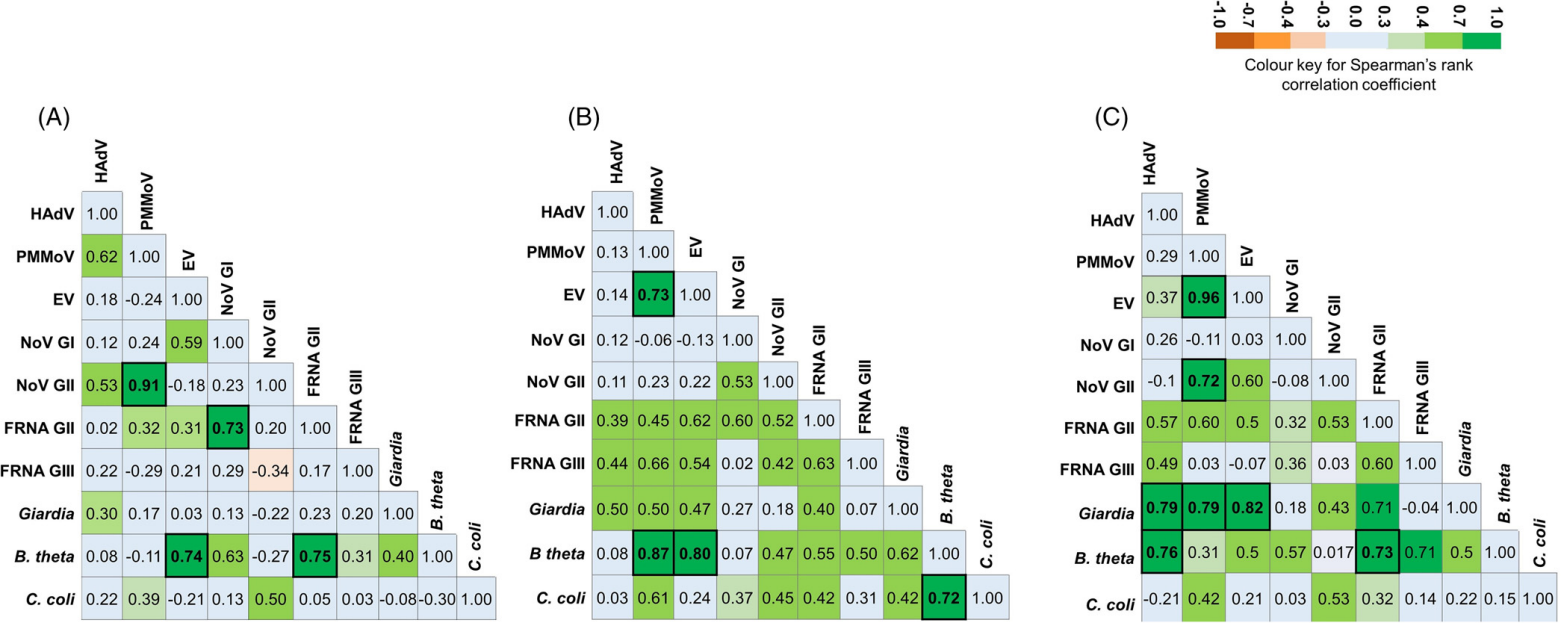
Correlation between microbial loads in influents, effluents, and log_10_ reduction values. Correlation (Spearman’s rank correlation coefficient) matrices of the microbial nucleic acid loads present in the (A) influent, (B) final effluent, and (C) log_10_ reduction values in the final effluent wastewater. A statistically significant correlation (*P* value ≤ 0.05) is indicated by bold font and an outline.

In order to understand the correlation, if any, among the concentrations (loads) of microbial indicators and the human pathogens detected in the influent, statistical correlations were computed. The Spearman’s correlation coefficient (ρ) was calculated between the nucleic acid loads (gc/L) of all of the microbial targets present in the influent samples to the final effluents and the LRVs in the final effluents (Fig. 5). Interestingly, the PMMoV (a virus indicator of human fecal contamination) loads showed a strong correlation to the NoV GII (a human pathogen) loads (ρ = 0.91, *P ≤ *0.05) in the influent samples. Similarly, the F^+^RNA GII (a bacteriophage indicator of human fecal contamination) loads showed a strong correlation to the NoV GI (a human pathogen) loads (ρ = 0.73, *P ≤ *0.05) among influent samples. *B. theta* (a bacterial indicator of human fecal contamination) loads correlated strongly with EV (human pathogens) loads (ρ = 0.74, *P ≤ *0.05) in influent samples tested. Among the three microbial indicators of human fecal contamination, the PMMoV loads did not have any correlation to the F^+^RNA GII loads or to the *B. theta* loads in the influent samples. However, the F^+^RNA GII loads and the *B. theta* loads showed a strong correlation with one another (ρ = 0.75, *P ≤ *0.05) (Fig. 5A).

Our data demonstrate nonuniform reduction in the loads of microbial indicators and human pathogens in the final effluents. In the final effluents, the PMMoV loads demonstrated a strong correlation with EV (ρ = 0.73, *P ≤ *0.05). The *B. theta* loads correlated strongly with PMMoV (ρ = 0.87, *P ≤ *0.05), EV (ρ = 0.80, *P ≤ *0.05), and C. coli (Fig. 5B).

The Spearman’s rank correlation was also used to determine significant relationships between the LRVs of microbial indicators and human pathogens in the final effluents. The results indicated that the reduction of PMMoV loads (LRV) correlated strongly with the LRVs of the EV loads (ρ = 0.96, *P ≤ *0.05), NoV GII loads (ρ = 0.72, *P ≤ *0.05), and Giardia loads (ρ = 0.79; *P ≤ *0.05) in the effluent samples. The extent of reduction (i.e., the LRV) in the Giardia loads in the effluent samples correlated strongly with the reductions in HAdV, PMMoV, EV, and F^+^RNA GII (ρ = 0.79, 0.79, 0.82, and 0.71, respectively; *P ≤ *0.05). The reduction in the *B. theta* loads showed a strong correlation with the reduction of the F^+^RNA GII loads (ρ = 0.73, *P ≤ *0.05) (Fig. 5C).

## DISCUSSION

These findings highlight the advantages of rapid screening for multiple microbial indicators of human fecal contamination/human pathogens by using real-time PCR to assess the influents and final effluents from WWTPs. Nonetheless, PMMoV and Giardia appear to be the best microbial markers of treatment performance in Indian settings as (i) they are consistently present at a high abundance (higher loads), (ii) their loads are less variable (low coefficient of variance) within and across the sites, and (iii) they show a strong correlation with other microbial indicators of human fecal contamination and human pathogens in both the influent and the final effluent samples.

The small number of samples and sites studied represents a major limitation of this study. In addition, most of the samples were collected during fall and thus may not capture seasonal variations, if any exist. This study quantified 12 microbial agents, including nonpathogenic indicators and human pathogens, including bacteria, enteric RNA viruses, an enteric DNA virus, bacteriophages, and a protozoan parasite from WWTPs in India for the first time. However, the quantitation of microbial genome copies in wastewater may not necessarily indicate the presence of infectious agents or a risk to human health. Nonetheless, our study demonstrates the advantages of molecular approaches for the rapid evaluation of multiple microbial targets in order to identify suitable microbial water quality targets for the safe and sustainable reuse of water in India (53).

### Correlation between microbial loads and physiochemical properties.

**FIG 6:**
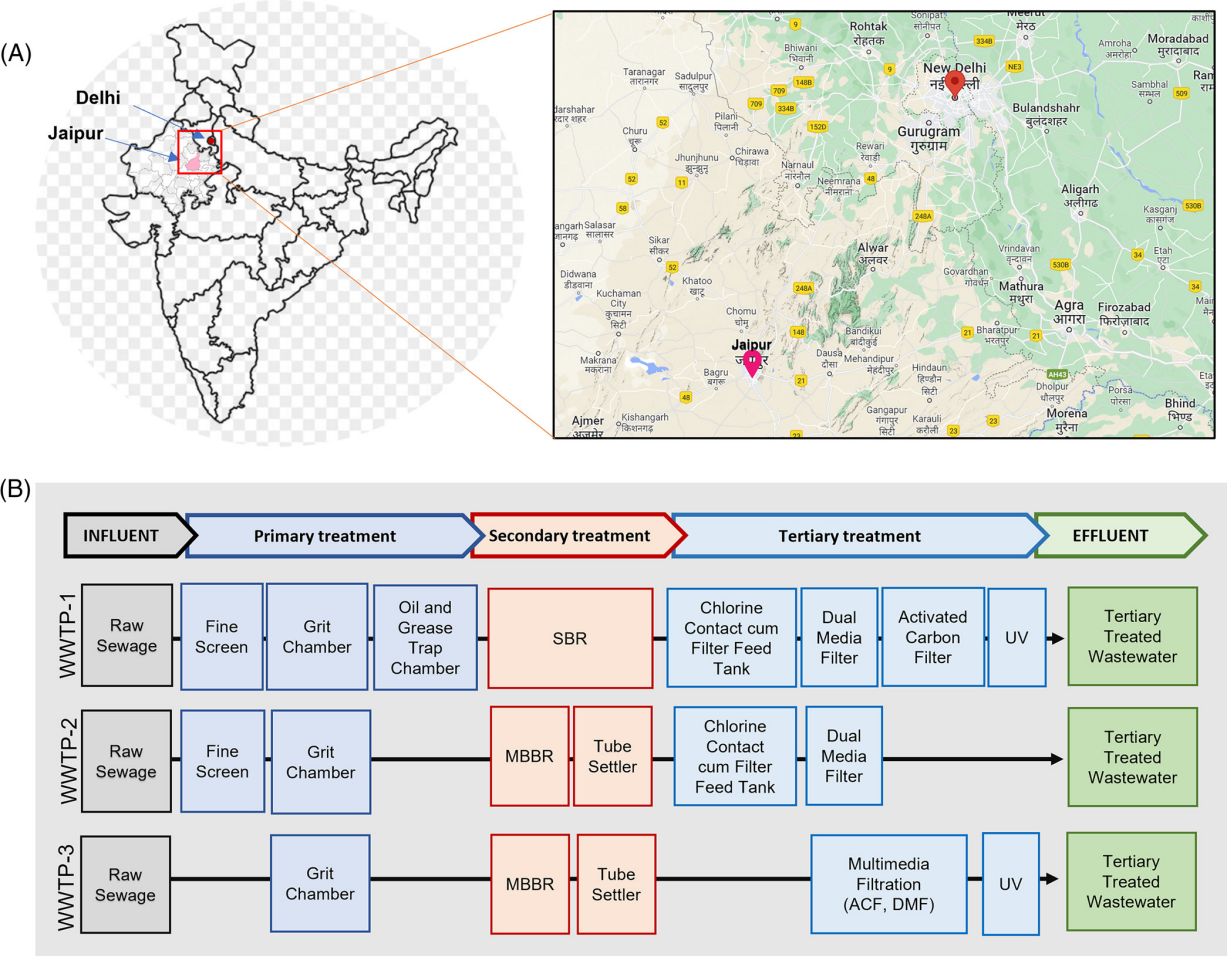
Sampling sites and wastewater treatment processes: (A) the wastewater samples were collected from wastewater treatment plants located in two major cities in northern (Delhi) and north-western (Jaipur) India, indicated on a map of India with a (inset) topographic view. (B) The layout of the treatment process employed at the wastewater treatment plants: WWTP-1 (Delhi), WWTP-2 (Jaipur, Rajasthan), WWTP-3 (Jaipur, Rajasthan). SBR, sequencing batch reactor; MBBR, moving bed biofilm reactor; DMF, dual media filter; ACF, activated carbon filter; UV, ultraviolet light.

Overall, there was no correlation between the microbial loads/LRVs and the physiochemical parameters evaluated in this study, with a few exceptions (Fig. S4; shown in bold font). For example, statistically significant correlations were found between the Giardia loads in the effluent and the BOD5 and COD levels in the effluent. However, this trend was not observed for the LRV of Giardia. As wastewaters harbor complex combinations of substances that are often unknown and have interactive effects (54), the poor correlation between the microbial loads/LRVs and the physiochemical parameters observed in this study may also be due to nonmicrobial factors that affect the physiochemical parameters, including the presence of fossil organic carbon (55). In addition, the small sample size or the differences in the wastewater treatment processes between the sites may also contribute to the weak correlation observed (Fig. 6B).

### Conclusion.

Through the comparative molecular quantification of 12 putative microbial pathogens and indicators of water quality for reuse applications in India, 2 microbial targets have been identified as suitable indicators of treatment performance. The findings of this research suggest that PMMoV and Giardia may be suitable microbial indicators of wastewater treatment processes in Indian settings, as they were consistently detected in the influent and/or effluent samples within and across the WWTPs, showing significant correlations between the log_10_ reduction patterns of human pathogens, such as noroviruses, adenoviruses, and enteroviruses. The consistent detection of several human pathogens, including enteric viruses and Giardia in the final effluents, highlights the potential risk associated with water reuse. This work lays the foundation for subsequent research related to microbial indicators and pathogens in recycled waters intended for reuse in India, in which seasonality, multiple cities, and additional microbial targets are considered in order to elucidate the performance of both microbial indicators and wastewater treatment processes that are meant to ensure the protection of the environment and public health.

## MATERIALS AND METHODS

### Study sites and sampling.

Three wastewater treatment plants, namely, WWTP-1 (located in south Delhi), WWTP-2 (located in Jaipur, Rajasthan), and WWTP-3 (located in Jaipur, Rajasthan) were included in this study. A total of 27 wastewater samples were collected for this study. From each WWTP, samples were collected on three separate occasions (i.e., on August 24, 2021, August 31, 2021, and September 7, 2021). For each sample, composite samples of wastewater were collected in carboys from different stages of treatment within each WWTP, including the influent raw sewage, the effluent after secondary treatment, and the effluent after tertiary treatment. The water samples were brought to the laboratory in an insulated cooler box as per the APHA guidelines (56) and processed for further analysis within 1 h of collection. Table 2 lists the WWTP characteristics and the details regarding the sample volumes. A description of the treatment processes employed at each WWTP is depicted in Fig. 6.

**TABLE 2:** Wastewater treatment plant (WWTP) characteristics and sample volumes

WWTP	Site of installation	Population served	Type of influent/sewage processed	Capacity	Flow rate	Reuse application	Sampling		
							Influent	2° effluent	3° effluent
WWTP-1	South Delhi	12,000 to 13,000	Institutional & Domestic waste	0.5 MLD	500 m^3^/day	Landscape irrigation	1L (*n* = 3)	10L (*n* = 3)	10L (*n* = 3)
WWTP-2	Jaipur, Rajasthan	10,000 to 11,000	Institutional waste	1.2 MLD	1,000 m^3^/day	Landscape irrigation	1L (*n* = 3)	10L (*n* = 3)	10L (*n* = 3)
WWTP-3	Jaipur, Rajasthan	9,000 to 10,000	Mixed (Municipal, hospital)	1.0 MLD	1,000 m^3^/day	Landscape irrigation	1L (*n* = 3)	5L (*n* = 3)	5L (*n* = 3)

### Physicochemical parameters.

The physicochemical parameters, including pH, water temperature, turbidity, BOD_5_, chemical COD, and TKN were monitored for all samples using standard protocols. Briefly, the pH was measured using a pH meter (57). Turbidity was measured using a Lutron turbidity meter (Model: TU-2016). The organic strength of the wastewater was measured by the 5-day BOD (BOD_5_) and COD, using standard methods (58) and the closed reflux method (59), respectively. TKN was analyzed using an infrared digestion unit (Turbotherm, Gerhardt, Konigswinter, Germany) after distillation in a semiautomatic steam distillation unit (Vapodest 20, Gerhardt, Germany).

### Primary and secondary concentration of wastewater samples.

The wastewater samples were subjected to DEUF, using Ashai Kasei REXEED-25S ultrafilters (Asahi Kasei Medical America Inc., Glenview, IL) as previously described (60), with a Cole-Parmer Masterflex L/S peristaltic pump (model D600 DS) (Cole Palmer, Vernon Hills, IL) and Masterflex neoprene silicone tubing (Masterflex) at a flow rate of 1 L/min. Autoclaved tubes and connectors were used in each analysis. After filtration, each REXEED-25S ultrafilter was backflushed with sterile 200 to 300 mL eluent (0.01% NaPP, 0.5% Tween 80, 0.05 M glycine; pH 9.0 to 9.5), and the eluate (primary concentrate) was collected in sterile polypropylene bottles. The eluate was further concentrated via polyethylene glycol (PEG8000) and sodium chloride (NaCl) precipitation. To each sample, we added PEG8000 (14%) and NaCl (0.2 M), and we kept the bottles for overnight vigorous shaking (4^°^C, 16 h), followed by centrifugation at 8,500 × *g*. The supernatant was carefully removed, and the pellet was resuspended in 8 to 12 mL of sterile phosphate buffered saline (PBS, pH 7.5) containing 0.25% Tween 80 (secondary concentrate).

### Nucleic acid extraction and quantitative evaluation of the microbial indicators.

Total nucleic acid from the secondary concentrates was extracted with two commercially available kits: a QIAamp UltraSens Virus Kit (Qiagen, GmbH, Germany) and an All Prep DNA/RNA Minikit (Qiagen, GmbH, Germany) for maximum recovery, following the manufacturer’s instructions. For every sample, an equal volume of nucleic acid extracts from each kit was combined, and 3 μL of the pooled extract was used as a template for each reaction. Real-time reverse transcription quantitative PCR (RT-qPCR) was performed for the quantitative evaluation of the RNA viruses (PMMoV, enterovirus, GI and GII norovirus, and F^+^RNA phages genogroups I and IV), whereas qPCR was performed for the quantitative evaluation of adenovirus, Giardia, *B. theta* and C. coli. The qPCR assays were performed using the 1-step QuantiNova Probe RT-PCR Kit (Qiagen, GmbH, Germany) in a 20 μL reaction volume with final concentrations of primers and probes corresponding to 0.8 μM and 0.25 μM, respectively. The primers and probes used in this study are listed in the Supplemental Material (Table S2).

### Generation of standard curves and quantitation of microbial nucleic acid loads.

The qPCR amplified products from raw sewage (influent wastewater) for each of the targets (i.e., adenovirus, PMMoV, enterovirus, norovirus GI, norovirus GII, Giardia, FRNA GII, FRNA GIII, *B. theta*, and C. coli) were purified and cloned into the pGEM T-easy vector and sequenced to ensure accuracy. Standard curves were plotted using log_10_ serial dilutions of the cloned plasmid DNA ranging from 10^5^ to 10^1^ copies/rxn in triplicate. The amplification efficiency was inferred via a linear regression analysis. All 9 of the real-time PCR assays were optimized to greater than 98% efficacy (R^2^ > 0.98). The concentration (microbial nucleic acid load) of each target organism was extrapolated from the respective standard curves and is indicated as genome copies/L (gc/L). The data (gc/L) was log_10_-transformed to obtain a normal distribution. The log reduction values (LRV) of the viruses that were achieved via the treatment processes were calculated using the following equation (61):

(1) $$\text{Log reduction value }\left(\text{LRV}\right)={\text{ Log}}_{\text{1}0}\text{ influent concentration }-{\text{ Log}}_{\text{1}0}\text{ effluent concentration}$$

### Statistical analysis.

The coefficient of variance (CV%) was calculated to assess the variability in the abundance of microbial targets (nucleic acid loads) across the three WWTPs, using the formula CV% = (Standard deviation/mean) × 100 in Microsoft Excel 2021 for Windows 10 (Microsoft Corp., Redmond, WA). Spearman’s rank correlation (ρ) test was performed to determine the association, if any, between the microbial nucleic acid loads present in the (i) influents, (ii) final effluents, and (iii) LRVs of the final effluent wastewaters. *P* values of* *≤0.05 were considered to be indicative of a statistically significant result. Scatterplots for the representation of the distribution of the microbial nucleic acid loads between and within the WWTPs, the ρ values, and the *P* values were calculated using GraphPad Prism version 9.0.
