# Supplementary material for: Quantitative Assessment of Microbial Pathogens and Indicators of Wastewater Treatment Performance for Safe and Sustainable Water Reuse in India
Source: Microbiol Spectr. 2022 Oct 31;10(6):e01720-22. doi: 10.1128/spectrum.01720-22 (PMC9769927; doi:10.1128/spectrum.01720-22)
Supplement: Supplemental file 1 — Supplemental material. Download spectrum.01720-22-s0001.pdf, PDF file, 0.8 MB [file spectrum.01720-22-s0001.pdf]

SUPPLEMENTARY INFORMATION

Quantitative assessment of microbial pathogens and indicators of wastewater treatment performance for safe and sustainable water reuse in India

Shruti Chowdhari[a], Shubham Rana[b], Samridhi Rana[c], Christina M. Morrison[e], Sarah Elizabeth Abney[e], Rajveer Singh[d], Patrick L. Gurian[d], Amit Kumar[b], Arun Kumar[c], Walter Q. Betancourt[e]#, Perumal Vivekanandan [a]#

[a]Kusuma School of Biological Sciences, Indian Institute of Technology Delhi, New Delhi, India

[b]Department of Civil Engineering, Malaviya National Institute of Technology, Jaipur, Rajasthan 302017, India

[c]Department of Civil Engineering, Indian Institute of Technology, Delhi, India

[d]Department of Civil, Architectural and Environmental Engineering, Drexel University, 3141 Chestnut Street, Philadelphia, PA, 19104, USA

[e]University of Arizona, Water & Energy Sustainable Technology (WEST) Center, 2959 W. Calle Agua Nueva, Tucson, AZ 85745, USA

E. mail: vperumal@bioschool.iitd.ac.in, wbetancourt@arizona.edu

Table S1: Physico-chemical parameters of the wastewater samples from WWTPs.

	Samples		pH	Water Temperature (^0C)	Turbidity (NTU)	BOD5 (mg/L)	COD (mg/L)	TKN (mg/L)
WWTP-1	Set-1	Influent	6.94	28.4	64	192	295	25.4
		2^0 Effluent	7.63	26.9	10.8	83	146	13.74
		3^0 Effluent	7.23	26.3	7.9	15	45	12.67
	Set-2	Influent	6.88	28.9	113	198	298	38.4
		2^0 Effluent	7.44	28.2	11.8	80	148	16.1
		3^0 Effluent	7.12	27.9	8.2	18	47	15.4
	Set-3	Influent	8.11	28.7	97	214	295	28.6
		2^0 Effluent	6.92	27.2	10.1	101	143	14.7
		3^0 Effluent	6.96	26.9	7.1	17	49	12.9
WWTP-2	set-4	Influent	6.6	23	25	104	414	122
		2^0 Effluent	7.6	24	9	70	180	97
		3^0 Effluent	8.1	24	11	35	69	67
	Set-5	Influent	7.7	30	32	92	490	132
		2^0 Effluent	7.4	29	11	75	155	74
		3^0 Effluent	7.6	29	16	42	78	54
	Set-6	Influent	7.7	29	35	121	435	115
		2^0 Effluent	7.3	29	16	64	105	85
		3^0 Effluent	7.3	30	12	40	61	71
WWTP-3	set-7	Influent	7.1	30	48	113	424	94
		2^0 Effluent	7.8	29	3	87	212	72
		3^0 Effluent	7.7	29	3	34	74	54
	Set-8	Influent	7.2	30	55	124	415	105
		2^0 Effluent	7.8	30	7	89	225	79
		3^0 Effluent	7.7	29	4	41	64	58
	Set-9	Influent	7.1	30	42	119	403	99
		2^0 Effluent	7.5	29	2	97	201	75
		3^0 Effluent	7.6	30	3	52	69	52

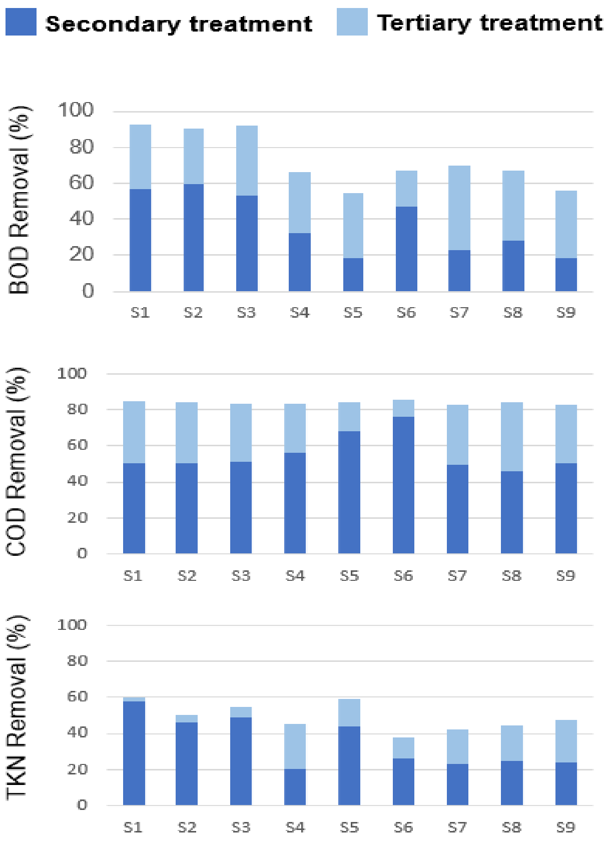

Fig S1: Percentage reduction in BOD5, COD and TKN in the effluent wastewater samples from all the three WWTPs included in the study. The samples S1-3 are effluents from WWTP-1, S4-6 are effluents from WWTP-2 and S7-9 are effluents from WWTP-3.

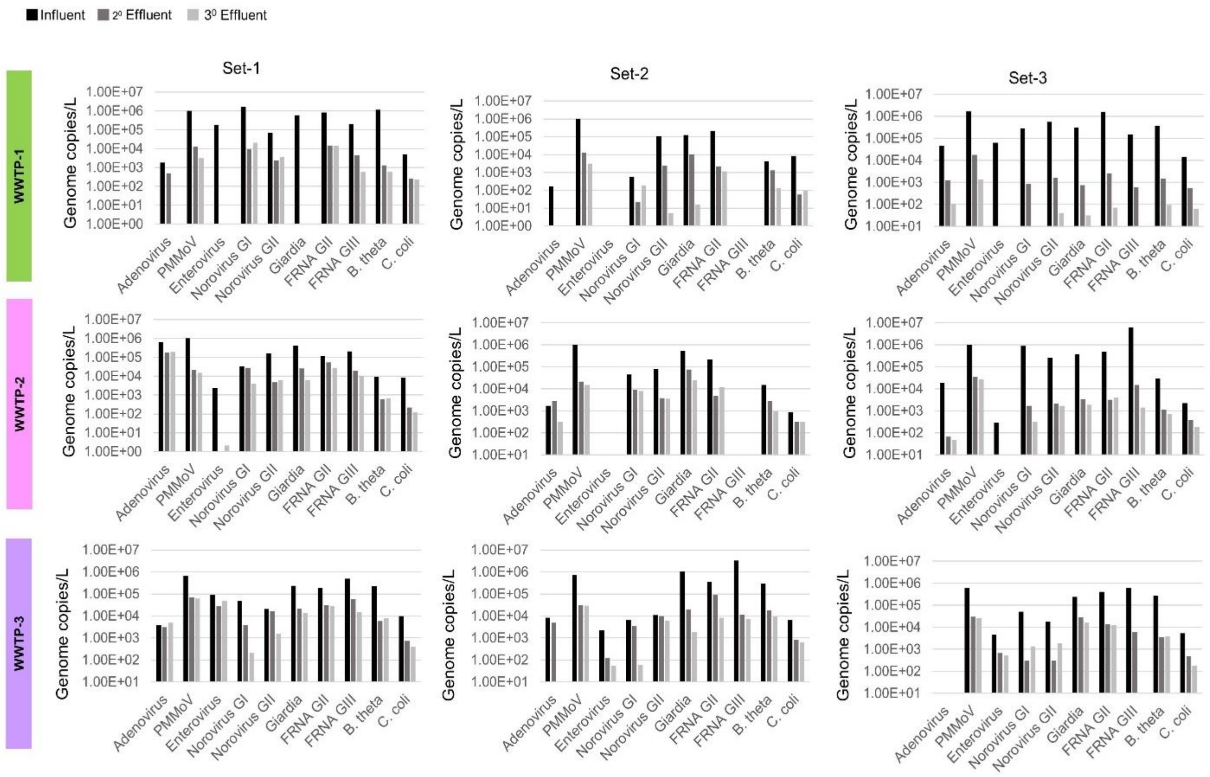

Fig S2: Concentration (gc/L) of microbe (HAdV, PMMoV, EV, NoV GI, NoV GII, *Giardia*, FRNA GII, FRNA GIII, *B.theta*, *C. coli*) in wastewaters collected at different stages of treatment including: influent, secondary effluent and tertiary effluent at 3 sampling time points from each WWTP.

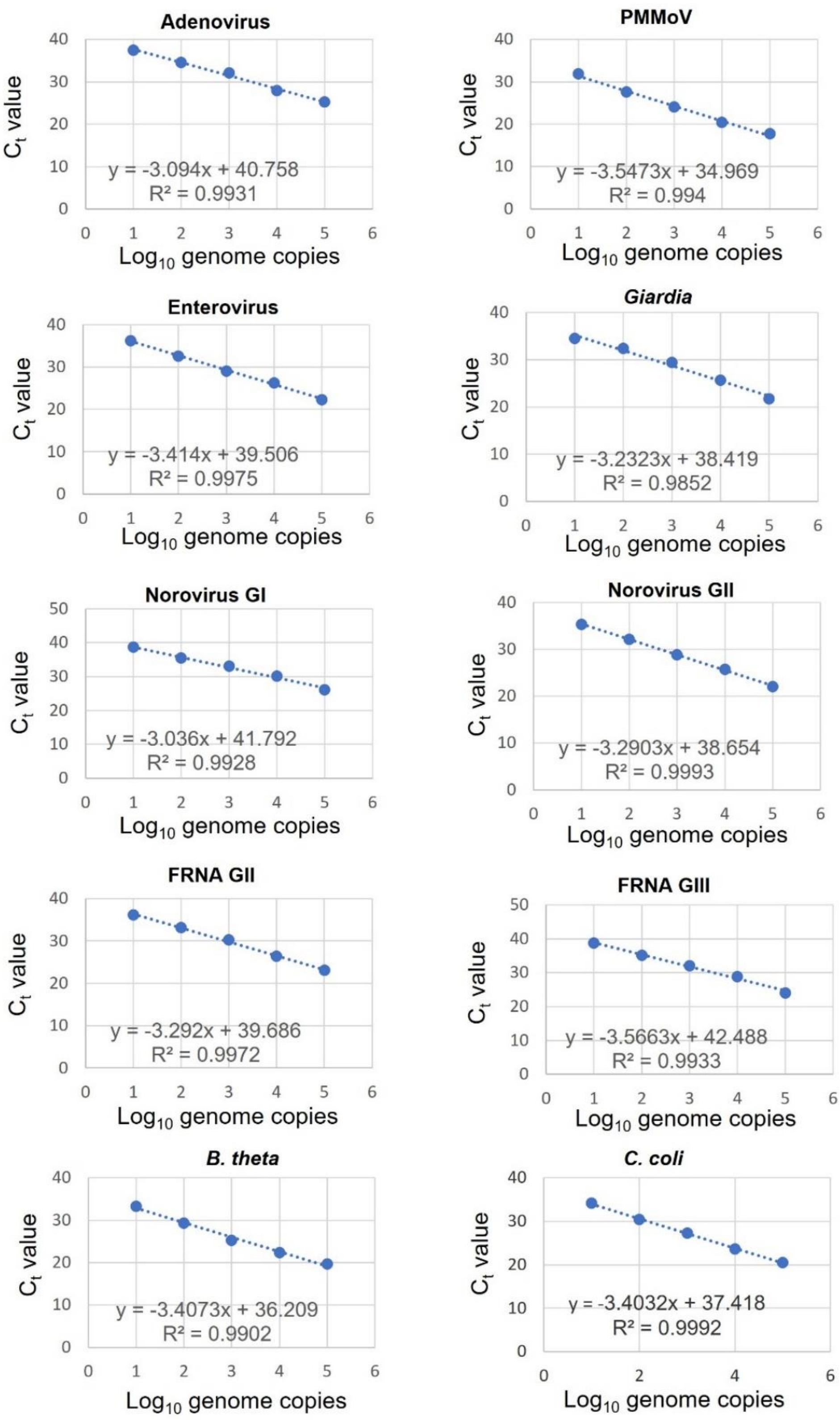

Fig S3: Standard curves generated by using 10-fold serial dilutions of pGEM-T easy plasmid containing the qPCR target gene fragment (62-143bp) for each microorganism obtained from influent wastewater nucleic acids tested in this study. Mean threshold cycle values (Ct) plotted against $\log_{10}$ genome copy number/qPCR reaction.

	Influents				Effluents				Reduction in effluents			
	Turbidity	BOD5	COD	TKN	Turbidity	BOD5	COD	TKN	Turbidity	BOD5	COD	TKN
**Adenovirus**	-0.23	0.03	0.03	0.17	0.28	0.00	0.61	0.42	-0.11	0.49	**0.73**	0.23
**PMMoV**	-0.09	0.18	0.00	0.14	-0.32	0.56	0.58	**0.68**	-0.44	0.52	0.31	**0.70**
**Enterovirus**	0.21	0.19	-0.50	-0.62	**-0.74**	0.40	0.55	0.28	0.11	0.49	0.26	0.13
**Norovirus GI**	-0.02	0.17	-0.20	-0.20	0.40	0.13	0.19	0.00	0.37	0.40	-0.02	-0.38
**Norovirus GII**	-0.02	0.23	-0.20	0.00	0.15	0.32	0.25	0.38	-0.57	0.37	0.18	0.18
**FRNA GII**	0.40	0.62	-0.43	-0.48	-0.24	0.10	0.49	0.17	0.00	0.63	0.42	0.23
**FRNA GIII**	-0.26	-0.08	0.28	0.16	-0.18	-0.07	0.25	0.57	0.48	0.10	-0.09	-0.22
Giardia	-0.28	-0.15	0.15	0.28	0.03	**0.80**	**0.95**	0.49	-0.10	0.78	0.58	0.43
B. theta	0.37	0.43	-0.44	-0.57	-0.39	**0.68**	**0.70**	0.50	0.45	0.57	0.17	0.27
c. coli	0.38	0.33	-0.45	-0.42	0.05	0.43	0.51	0.3	0.15	0.43	-0.45	0.12

Fig. S4: Correlation between microbial loads in influents, effluents and $\log_{10}$ reduction values (LRV) with respective physiochemical parameters: Correlation (Spearman's rank correlation coefficient) matrices of the microbial nucleic acid loads in the (a) Influent, (b) final effluent and the (c) LRV in the final effluent wastewater with physiochemical properties. Significant correlation (*p value* ≤ 0.05) is indicated in bold font.

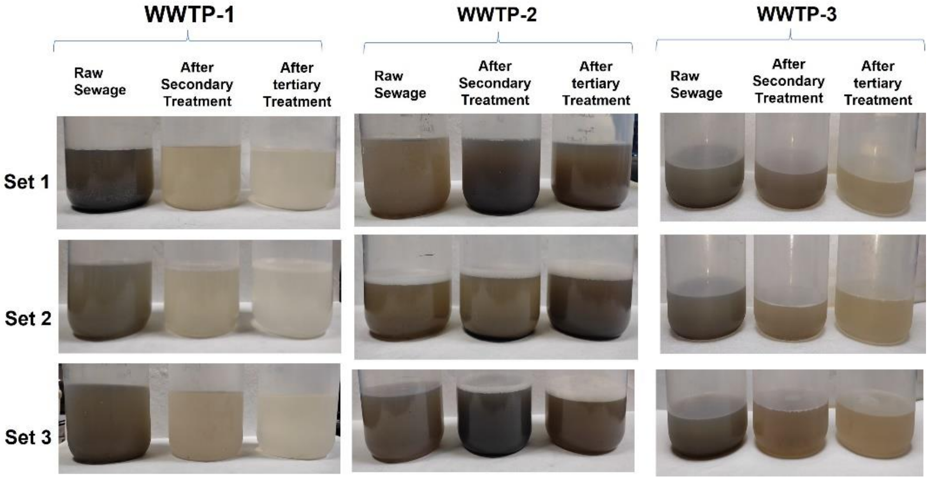

Fig S5: Pictures of the primary concentrates recovered after Dead End Ultra filtration using Rexeed 25S ultra-filters.

Table S2: List of qPCR primers and probes

Micro bial targets	Primer or probe	Sequence (5’→3’)	Length of amplicon	Target gene description	Reference
Adenovir us	AQ2 f<br>AQ1r<br>AP	GCCCCAGTGGTCTTACATGCACATC<br>GCCACGGTGGGGTTTCTAAACTT<br>FAM-TGCACCAGACCCGGGCTCAGGTACTCCGA-HQ1	131	Hexon protein gene	(Heim et al., 2003)
PMMoV	PMMoV FP<br>PMMoV RP	AGTGGTTTGACCTTAACGTTTGAG<br>TTCCTCCTTTGATGTAATTGTC	84	Replication associated protein	This study.
Enterovir uses	EV1F<br>EV1R<br>EV-P	CCCTGAATGCGGCTAAT<br>TGTCACCATAAGCAGCCA<br>FAM-ACGGACACCCAAAGTAGTCGGTTC-BHQ1	143	5’UTR of poliovirus, Pan-enteroviral primers	(Gregory et al., 2006)
GI Noroviru s	COG1F<br>COG1R<br>RING1(b)-TP	CGYTGGATGCGNTTYCATGA<br>CTTAGACGCCATCATCATTYAC<br>FAM-AGATCGCGGTCTCCTGTCCA-BHQ1	85	RdRP	(Kageyama et al., 2003)
GII Noroviru s	COG2F<br>COG2R<br>RING2-TP	CARGARBCNATGTTYAGRTGGATGAG<br>TCGACGCCATCTTCATTCACA<br>FAM-TGGGAGGGCGATCGCAATCT-BHQ1	98	RdRp	(Kageyama et al., 2003)
Giardia	G-F<br>G-R<br>G-p	GACGGCTCAGGACAACGGTT<br>TTGCCAGCGGTGTCCG<br>CCCGCGGCGGTCCCTGCTAG	62	18s rRNA	(Verweij et al., 2003)
FRNA GI	FRNA GI-F<br>FRNA GI-R<br>FRNA GI -p	GTCCTGCTCRACTTCCTGT<br>CGGCTACCTACAGCGATAG<br>FAM-CAWGGTAGCGTCTCGCTAAAGACATTA-BHQ1	82	Strain: MS2 (NC 001417)<br>Primer binding site: 20-102 bp.	(Wolf et al., 2008)
FRNA GII	FRNA GII-F<br>FRNA GII-R<br>FRNA GII -p	TCTATGTATGGATCGCACTCG<br>GTAGGCAAGTCCATCAAAGTC<br>TGCTGTCCGATTTCACGTCTATCTTCA	111	Strain: GA (NC 001426.1)<br>A (gene description: terminase large subunit family protein)	(Wolf et al., 2008)
FRNA GIII	FRNA GIII -F<br>FRNA GIII -R<br>FRNA GIII -p	GYGGTGCYACAACRACGAAT<br>GWGGSGTACACKCTTGCG<br>TACGGYCATCCGTCCTTCAAGTTTG	77	Strain: Qβ (AY099114.1)<br>Q beta replicase	(Wolf et al., 2008)
FRNA GIV	FRNA GIV-F<br>FRNA GIV-R<br>FRNA GIV -p	GACWGGTCGGTACAAAGTKG<br>ARCTTCACCTCGGGAARTC<br>FAM-CGGATGAAGGCACTGTCCTGAATC-BHQ1	86	Strain: SP (X07489.1) unnamed protein product; replicase beta-subunit (AA: 1 - 576)	(Wolf et al., 2008)
B. theta	BtH-F<br>BtH-R<br>BtH-P	CATCGTTCGTCAGCAGTAACA<br>CCAAGAAAAAGGGACAGTGG<br>FAM-ACCTGCTG-NFQ	62	α-1-6 mannanase	(Yampara-Iquise et al., 2008)
C. coli	C. coli FP<br>C. coli RP	GAAGTATCAATCTTAAAAAGATAA<br>AAATATATACTTGCTTTAGATT	72	16S-23S rDNA internal transcribed spacer (ITS) region	(Khan IUH *al.*2007)
